# Serum TSH and Daily Physical Activity in a Cohort of Nonagenarians: Results from the Mugello Study

**DOI:** 10.3390/jfmk7030056

**Published:** 2022-08-05

**Authors:** Andrea Di Blasio, Giulia Di Dalmazi, Pascal Izzicupo, Ines Bucci, Cesidio Giuliani, Angela Di Baldassarre, Francesca Cecchi, Raffaele Molino Lova, Federica Vannetti, Giorgio Napolitano, Claudio Macchi

**Affiliations:** 1 Department of Medicine and Aging Sciences, “G. D’Annunzio” University of Chieti-Pescara, Via L. Polacchi, 11, 66100 Chieti, Italy; 2 IRCCS Fondazione Don Carlo Gnocchi, Via di Scandicci, 269, 50143 Firenze, Italy; 3 Department of Experimental and Clinical Medicine, University of Firenze, P.za di San Marco, 4, 50121 Firenze, Italy

**Keywords:** thyroid hormones, elderly, physical activity

## Abstract

Background. The current literature does not furnish clear data concerning the relationship between thyroid function, sedentary time and daily physical activity (PA) in older adults with euthyroid condition. The aim of this study was to investigate the association of serum Thyrotropin-Stimulating Hormone (TSH), free Triiodothyronine (fT3) and free Thyroxine (fT4) with sedentary time and PA in a cohort of nonagenarians. Methods. A total of 108 nonagenarians (92.8 ± 3.2 years), participating in the Mugello Study, and with complete data on thyroid function, sedentary time, PA and sleeping (objectively collected through a multisensory device), were considered for the analysis. Results. Mainly, TSH negatively correlated with time spent lying down, and positively correlated with METs. fT4 levels were negatively associated with mean daily metabolic equivalents (METs) and with low-intensity PA practice (LIPAT), and positively associated with lying down and sleeping time. Similar results have been shown in the female sample. Mainly, participants with high-normal (third tertile) versus low-normal TSH (first tertile) had higher moderate-intensity PA (MIPAT) (*p* = 0.03). In the female sample, first TSH tertile had higher METs (*p* = 0.010), LIPAT (*p* = 0.02), MIPAT (*p* = 0.01) and lower time lying down (*p* = 0.04) than third TSH tertile. Conclusion. High-normal serum TSH and low-normal fT4 are associated with higher levels and intensity of daily PA, together with higher MIPAT continuity, in the oldest-old.

## 1. Introduction

Human aging represents a challenge for the normal function of the hypothalamic-pituitary-thyroid (HPT) axis [1]. The interaction between thyroid function and aging has been examined in a recent review [2] and reported data are somewhat conflicting. Indeed, notwithstanding, some studies have reported a decrease in serum Thyrotropin-Stimulating Hormone (TSH) with aging [3,4], others have shown no change in TSH levels related to aging [5,6]. However, the majority of studies revealed that serum TSH gradually increases with age [7,8]. The same uncertainty also pertains to thyroid hormones, which have been shown to decrease simultaneously or to behave separately, with a decline in free Triiodothyronine (fT3) [9,10,11] and an increase in free Thyroxine (fT4) [3,12,13]. An increase in serum reverse T3 concentrations with age, partially independent from non-thyroidal illness, has also been reported [14]. The described changes have been interpreted as an adaptive response to energy resources and demands [15].

Relatively recent data demonstrated that thyroid function variations, even within the euthyroid reference range, may be associated with adverse health outcomes in older adults, including all-cause mortality [16,17]. Once again, data are conflicting: increased all-cause mortality has been associated with an increase [18,19] or decrease [17,20,21] in TSH levels, as well as an increase [7,16,18,22,23,24], or decrease [25] in fT4 values. Inconsistency of data can be, at least partially, explained by the selection of patients; indeed, different age, sex ratio, hospitalized vs. home-living, and inclusion or exclusion of patients with non-thyroidal illness have to be considered.

In the search of parameters of thyroid function correlated with healthy aging, of particular interest, in our opinion, is the observation that low-normal TSH levels have been associated with an increased risk of frailty [26] and hip-fracture in euthyroid women, but not in men, aged 65 years and more [27]. Contrarily, low-normal fT4 has been associated with better mobility, physical function and higher handgrip strength [28,29,30], while fT3 levels have been reported to correlate positively with physical performance scores in older euthyroid individuals [31].

These findings suggest that thyroid function variations, even within the normal range, may influence the quantity and quality of daily physical activity (PA) in older people as an adaptive response of bodily functions to maintain/favor their health. Indeed, functional mobility, fitness level and a more favorable fatigue profile also sustain PA practice in later life in a virtuous circle extending lifespan and reducing the risk of disability [28,32,33]. In our hypothesis, coupled with the variation in thyroid functions, within the normal range, there is the variation in PA and sedentary time to maintain bodily homeostasis and health according to the increase or decrease in thyroid functions. However, limited data are available on the relationship between thyroid function, daily PA and sedentary behavior patterns in older adults, especially in the oldest old, when PA is not influenced by family and occupational duties, but also when the health problems, linked with aging, and occupational life, may negatively affect daily movements.

Therefore, in the present study, we investigated whether serum TSH and fT4 were associated with sedentary time and daily PA, while also considering the dimensions of the latter (i.e., intensity, duration, frequency and volume) in a cohort of self-sufficient and non-hospitalized nonagenarians enrolled in the frame of an epidemiological study conducted in the Mugello area, Tuscany, Italy.

## 2. Materials and Methods

### 2.1. Study Population

The analysis presented in this paper is based upon data from the Mugello Study, a survey on nonagenarians living in the Mugello area, north-eastward of Florence, in Tuscany, Italy, whose detailed description is reported elsewhere [34].

Briefly, 470 (130M, 340F) non-selected nonagenarians living in the Mugello area underwent a structured interview and medical visit conducted by a trained physician at their residence, along with a series of physical, performance and instrumental assessments. The latter was aimed to characterize body composition, cardiovascular health, daily physical activity, sleep and strength of participants [34]. Blood samples were collected one week later by a nurse to perform routine lab tests and to create a biologic bank with serum and plasma aliquots. The “The Mugello Study: a lesson from older persons aged 90 years and over”, which complied with the principles of the Declaration of Helsinki on clinical research involving humans, was approved by the Ethics Committee of IRCCS Fondazione Don Carlo Gnocchi, ONLUS, Milano, Italia on 15 December 2010, and the participants, or their legal representative, signed the informed consent form to be included in the study, and to undergo blood withdrawal. Of the 470 nonagenarians, 108 (27M, 81F) were selected to be included in this study, as they were free from cognitive disabilities, previously diagnosed or clinically evident endocrine diseases, pharmacological treatments concerning and/or affecting thyroid function, and from any other disease negatively affecting daily movement. Selected participants were independent in all of the activities of daily living (ADL). Frequencies of diseases affecting participants are shown in Table 1.

### 2.2. Anthropometric Measures

Weight, height, ulna length, lower limb length, abdominal and hip circumference, as well as maximal relaxed arm, thigh and leg circumference, were measured following the guidelines of the International Society for the Advancement of Kinanthropometry [35].

Body composition was assessed by using a body impedance assessment (BIA) (EFG, Akern, Italy). Fat mass index (FMI), free-fat mass index (FFMI) and muscle mass index (MMI) were calculated according to the following formulas: FMI = kg of fat mass/stature (m)^2^; FFMI = kg of free-fat mass/stature (m)^2^; MMI = kg of muscle mass/stature (m)^2^.

### 2.3. Sedentary Time and Daily Physical Activity (PA) Assessment

Daily physical activity was recorded under free-living conditions over seven consecutive days, including weekdays and weekend days, by using a SenseWear Armband (Body Media Inc., Pittsburg, PA, USA) applied to the participant over the triceps muscle, halfway between the acromion and the olecranon processes, as suggested by the manufacturer. This system integrates the information gathered by the two-axis accelerometer and sensors (i.e., skin and near-body temperature, heat flux, and galvanic skin response) with sex, age, stature, weight, smoking status and handedness of the user. The Armbands thus provide qualitative (e.g., intensity) and quantitative (e.g., number of daily steps, energy expenditure) information about daily PA [36]. We considered the following parameters: the mean daily metabolic equivalent (METs), sedentary time (activities with an intensity ≤1.5 METs), excluding lying down and sleeping, low-intensity PA (LIPAT, intensity >1.5 METs and ≤3 METs), moderate-intensity PA (MIPAT, intensity >3 METs and ≤6 METs), vigorous-intensity PA (VIPAT, >6 METs), daily lying down, sleeping and sleeping efficacy, calculated as ratio between sleeping to lying down ×100 [37]. The SenseWear Armband is able to discern between daily lying down and sleeping integrating the information concerning the heat flux, and galvanic skin response, with those furnished by the two-axis accelerometer. Indeed, during sleeping, heat flux and galvanic skin response have a different trend compared to simply lying down, even in the absence of movement. The participants wore their monitors all day, except while bathing. The wear time criteria, to consider valid registrations, were at least 540 min/day on weekdays and 480 min/day on weekend days [38]. We also calculated the number of daily bouts (B) of PA spent at MIPAT and VIPAT intensities lasting from 1 to 10 consecutive minutes, the sum of daily bouts lasting less than 5 consecutive minutes (B < 5), the sum of daily bouts that were major or equal to 5 (B ≥ 5) and 10 (B ≥ 10) consecutive minutes. Here, B < 5, B ≥ 5, and B ≥ 10 were calculated using a specifically written application. The application, as first action, calculated the number of bouts (B) of MIPAT and VIPAT, and their totals, which were gathered according to their consecutive durations (i.e., 1 consecutive minute, 2 consecutive minutes and so on until 10 consecutive minutes), then calculated the variables under consideration. Mean duration and intensity of bouts of PA spent at moderate and/or vigorous intensities were also calculated.

### 2.4. Thyroid Function and Inflammatory Status

One week after the medical interview and the clinical visit, a nurse visited the participant’s residence to draw blood samples for lab tests. Serum and plasma aliquots were stored at −80° in the biologic bank. TSH and fT4 fT3 were measured using a chemiluminescence method (normal values 0.34–5.60 mUI/mL, 2.00–3.90 pg/mL and 0.58–1.64 ng/dL for TSH, fT3 and fT4, respectively). High-sensitivity C-reactive protein (CRP) was measured in duplicate using an ELISA and colorimetric competitive immunoassay that used purified protein and polyclonal anti-CRP antibodies.

### 2.5. Statistical Analysis

Firstly, data were tested for normality using the SAS 9.2 software (SAS Institute Inc., Cary, NC, USA), and they are presented as means ±standard deviation. As the sample was composed by female (n = 81) and male (n = 27) participants, the Student’s *t*-test (for continuous variables) and the chi-square test (for categorical variables) were used to verify whether the sub-samples differed in the investigated variables. Due to the huge difference between samples sizes, the Student’s *t*-test results were verified through the Mann–Whitney’s test (data not shown), providing the same results. Considering the whole sample, Spearman’s correlation test was run adjusting for the effects of age, CRP and MMI, being well-known influencing factors of PA. After that, using the TSH variable as the clustering variable, we firstly gathered the sample according to the values of the TSH tertiles and then compared the three sub-samples’ characteristics using the one-way analysis of variance and Bonferroni test was used as post hoc analysis. First tertile included participants having 0.18 ≤ TSH < 1.11 μU/mL; second tertile included participants having 1.11 ≤ TSH ≤ 2.12 μU/mL; third tertile included participants having 2.12 < TSH ≤ 7.98 μU/mL. The same analysis was repeated separately in female and male subsamples. The female subsample had the same values of TSH tertiles of the whole group, while in men, the first tertile included participants having 0.73 ≤ TSH < 1.12 μU/mL; the second tertile included participants having 1.12 ≤ TSH ≤ 1.51 μU/mL; the third tertile included participants having 1.51 < TSH ≤ 7.96 μU/mL. A *p* ≤ 0.05 was considered statistically significant.

## 3. Results

### 3.1. Study Population Characteristics

Characteristics of the study population are shown in Table 1. The mean age was 92.84 ± 3.18 years. Generally, the 108 participants had a good body composition; both for female and male participants, FMI was near to the 25th percentile, while their FFMI ws near the 75th percentile of distribution, according to the existing reference values, with a central body fat distribution [39].

The analysis of daily PA data indicates a lifestyle characterized by high daily sedentary time and low daily moderate to vigorous PA practice. Indeed, participants were mainly engaged for about 11 h in sedentary activities, excluding daily lying down and sleeping time, while they meanly reserved about 3 h of their day to light PA practice, about 30 min to moderate, and 0 min to vigorous PA practice. Focusing our attention on the analysis of moderate PA organization, we show that notwithstanding the low mean time, the moderate PA of our sample was highly fractioned during the day. Indeed, participants had a high number of daily bouts of moderate PA, mainly composed by bouts lasting from 1 to 4 consecutive minutes, with a prevalence of bouts lasting 1 min (B1).

Mean TSH, fT3 and fT4 levels were 1.91 ± 1.48 μU/mL, 2.79 ± 0.36 pg/mL and 0.83 ± 0.18 ng/dL, respectively; all patients had normal fT3 and fT4 levels, while 3 patients (3 F) had TSH values between 0.18 and 0.34 UI/mL and 4 patients (1M, 3F) had values between 5.6 and 7.98 UI/mL. Due to the small difference from the normal range and the low number of patients with subclinical thyroid dysfunction, these 7 patients were not excluded from the study.

The observation of CRP values suggests a mean normal inflammatory condition, even if the range of distribution reveals the presence of participants having a systemic proinflammatory and inflammatory status.

When the sub-sample characteristics (i.e., female vs. male sub-samples) were compared, no significant differences for thyroid function, sleeping, sedentary and PA parameters were underlined (Table 1).

### 3.2. Thyroid Hormones Correlations in the Whole Sample

TSH and fT4 showed a significant inverse correlation overall (r = −0.29, *p* < 0.01).

The Spearman’s correlation test, after correction for the effects of age, CRP and MMI, well recognized influencing factors of both thyroid function and/or PA [28], showing that TSH has a positive correlation with bouts of moderate PA lasting 4 consecutive minutes (B4), and a negative correlation with lying down time. As far as fT4 concerns, positive correlations have been shown with lying down and sleeping time, while negative correlations have been found with METS, and LIPAT (Table 2). Bearing in mind that almost all patients were euthyroid, these results show that thyroid function, independently from age, CRP and MMI, is negatively correlated with daily movement in a sample of nonagenarian men and women. The results show that the higher the mean daily METs, LIPAT and B4 are, the less active thyroid function is. No significant correlations were found between serum fT3 levels, daily PA and sleeping parameters (Table 2).

### 3.3. Comparison according to TSH Tertile Membership

To deepen the partial correlation results, the sample was clustered, according to TSH value, in three sub-samples (Table 3). The one-way analysis of variance and Bonferroni test, used as post hoc analysis, showed that the three sub-samples significantly differed for MIPAT and B ≥ 5 as well as fT4 (obvious result, due to the fact that we used TSH as clustering variable). Specifically, the post hoc analysis showed that the nonagenarians being in the 1st tertile of TSH (0.18 ≤ TSH < 1.11 μU/mL) had lower MIPAT and B ≥ 5 compared to those in the 3rd tertile (2.12 < TSH ≤ 7.98 μU/mL).

### 3.4. Thyroid Hormones Correlations and Comparison according to TSH Tertile Membership in Female Sub-Sample

Since our sample was mainly composed of female nonagenarians (i.e., n = 81/108), we repeated the analysis in women and men separately. Table 4 shows the results of Spearman’s correlation test, ran adjusting for the effects of age, CRP and MMI, of the female sub-sample, confirming, with stronger data, those obtained from the whole sample. Indeed, TSH has been found to be strongly and positively correlated with METs, MIPAT, B4, and B ≥ 5, while fT4 is negatively linked to METs, LIPAT, MIPAT and B ≥ 5.

Furthermore, serum fT3 levels were positively correlated with METs in the female sub-sample (r = −0.22, *p* < 0.05).

The results observed in Table 3 were reinforced when the female sub-sample was clustered, according to TSH value, in three sub-samples, being compared through the one-way analysis of variance and Bonferroni test (Table 5). Female nonagenarians, being the 1st tertile of TSH (0.18 ≤ TSH < 1.11 μU/mL), had lower METs, LIPAT, MIPAT, B4 and B ≥ 5, and higher daily time spent lying down, compared to those in the 3rd tertile (2.12 < TSH ≤ 7.98 μU/mL).

### 3.5. Thyroid Hormones Correlations and Comparison according to TSH Tertile Membership in Male Sub-Sample

When the Spearman’s correlation test was repeated considering just the male sub-sample (n = 27/108), a correlation between TSH and fT4 (ρ = −0.400, *p* = 0.05) was found, while no significant differences in age, body composition, sedentary time, PA, sleeping and inflammation were found according to TSH tertile membership (data not shown).

## 4. Discussion

As a general view of PA in our female nonagenarians, meanly characterized by low FMI and high FFMI, none of them reached the recommended amounts of aerobic moderate PA, i.e., 30 min of moderate PA through multiple bouts lasting almost 10 consecutive minutes throughout the day [40]. Indeed, their daily moderate PA is mainly composed of bouts lasting from 1 to 4 consecutive minutes (Table 1). Even if this distribution is a weak point to reach and maintain cardio-metabolic health through PA [40], it helps to break sedentary time and to maintain physical functions. Indeed, there is increasing literature stating the importance of breaking up prolonged sitting time, especially in old people [41,42]: according to Sardinha and colleagues, functional disability in oldest-old adults is linked to very few breaks (less or equal to every 7 h) of sedentary time, independently of moderate-to-vigorous PA [42].

Focusing our attention on TSH and fT4 we found TSH positively correlated with B4_,_ and negatively correlated with lying down time. On the contrary, fT4 showed positive correlations with sedentary and lying down time and negative correlations with METS and LIPAT. These findings were confirmed if our population was clustered according to TSH tertiles, as higher values are linked to higher total daily MIPAT and more prolonged activity (i.e., B > 5 min). The most convincing data, however, were obtained if we considered only female nonagenarians. Indeed, in this more homogeneous group (which is also the most representative including 81/108 subjects, 75% of the sample), almost all the parameters of PA (namely METS, MIPAT, LIPAT, B4 and B > 5) were correlated with the classically considered “lower thyroid function”. Bearing in mind that almost all subjects were euthyroid, these results show that, in a sample of nonagenarians, thyroid function, independently from age, CRP and MMI, is negatively correlated with daily movement. The results show that the less active thyroid function is, the higher daily PA is. Our results underline the presence of a correlation among the variables without establishing a cause–effect relationship.

As there is limited and poor literature stating both that PA can modulate thyroid function, and that thyroid function can modulate PA in euthyroid persons, our results can be considered important evidence of the correlations of TSH in the field of non-exercise PA, and add a support to the theory about the role of TSH in daily PA modulation, at least in nonagenarians. Specifically, the existing literature concerning the role of PA on thyroid function, is based on studies not applicable to our design and population. Indeed, Hackney and Saeidi [43] studied the effects of short-term and intense physical exercise on serum TSH and thyroid hormone concentration in young adults. On the other hand, Dueñas et al. [44] failed to show significant association between thyroid function and PA, in euthyroid adults and seniors, even in the presence of both longitudinal and cross-sectional design, with a study including subjects with a wider range of age and using questionnaires. In addition, they used a validated questionnaire to estimate PA, which is well-known to be less sensitive than accelerometers [45], in participants younger than those used in its validation study. Conflicting data have been presented by Ravaglia et al. [46] and by Ceresini et al. [31], who showed opposite correlations of thyroid hormones and TSH with PA. However, these studies included a small number of participants [46], much younger than those included in the present study [31,46], and were mainly based on validated questionnaires [31,46]. Finally, the paper by Ceresini et al. [31] intended to study both spontaneous PA and exercise practice, while the present study concerns daily PA. Therefore, according to our results, in nonagenarian subjects, the more TSH is within the normal range, the more stable daily PA is for health, i.e., high mean daily METs and daily steps, low-fractioned MIPAT and low sedentary time. This also reinforces the suggestion that having a TSH closer to or slightly above the normal upper limit is a protective condition at this age.

Many conflicting reports are present in the literature about the correlation of thyroid function and health in aging people; it is generally accepted, however, that higher TSH and lower fT4 are associated with lower mortality in old subjects, especially centenarians. Moreover, lower mortality has been reported in parents of nonagenarian siblings, who showed higher serum TSH levels and lower fT4 levels [2]. As a putative explanation, aging is associated with decreased levels of THs, thus, according to the remodeling theory of aging, such a situation may represent an adaptive phenomenon to reduce basal metabolic rate and to prevent excessive catabolism, finally leading to “successful aging” [2].

In order to compare our results with those obtained in different studies, it has to be underlined that the subjects of the present study were free from cognitive disabilities, previously diagnosed or clinically evident endocrine diseases and independent in all of the ADL; finally, they were all from an iodine sufficient area [47]. As a limitation, our results were significant in the whole population and in the female subset, while the male subset did not confirm the same results, possibly due to the small number of subjects enrolled.

## 5. Conclusions

Our results describe the status of daily PA in healthy nonagenarians and its correlation with thyroid function. These findings may add information about optimal levels of thyroid hormones in the elderly, bearing in mind that almost all patients included in the study showed TSH and fT4 levels within the normal range, i.e., they were euthyroid according to common reference values. It follows that the observed correlations between thyroid function and PA in our population of nonagenarians cannot be extended to patients with subclinical hypothyroidism, but it is also implied that our results support the concept that a slight increase in TSH is associated with better functional mobility, fitness and a reduced risk of disability in the considered population. We cannot establish a cause–effect based on our data; however, according to a recent review showing no effect of physical exercise on thyroid function [48], it seems reasonable to suppose that a slight increase in TSH is beneficial to daily PA in female nonagenarians.

## Figures and Tables

**Table 1:** Characteristics of the study population and sub-samples differences.

Characteristics	Whole Sample (N = 108)	Female Sub-Sample (*n* = 81)	Male Sub-Sample (*n* = 27)	Female vs. Male Sub-Sample *p*
Age (years)	92.84 ± 3.18 (89–101)	93.07 ± 3.30 (89–101)	92.15 ± 2.74 (90–100)	0.192
Waist circumference (cm)	93.87 ± 11.40 (68–130)	93.82 ± 11.23 (68–125)	101.85 ± 9.84 (82–130)	0.001
Hip circumference (cm)	102.12 ± 9.96 (62–128)	100.99 ± 10.51 (62–128)	105.58 ± 7.17 (92–120)	0.040
Waist to Hip ratio	0.93 ± 0.06 (0.78–1.11)	0.93 ± 0.07 (0.78–1.11)	0.96 ± 0.05 (0.89–1.10)	0.040
FMI (kg of fat mass/m^2^)	6.76 ± 3.67 (0.04–16.33)	7.43 ± 3.78 (0.75–16.33)	4.76 ± 2.47 (0.04–10.17)	0.001
FFMI (kg of fat-free mass/m^2^)	18.51 ± 2.85 (13.08–26.40)	20.68 ± 2.78 (13.08–23.47)	17.79 ± 2.50 (16.34–26.40)	<0.001
MMI (kg of muscle mass/m^2^)	11.65 ± 2.80 (6.30–21.38)	11.05 ± 2.40 (6.30–17.73)	13.47 ± 3.14 (8.41–21.38)	<0.001
METs (METs)	1.01 ± 0.21 (0.65–1.66)	1.01 ± 0.22 (0.65–1.66)	1 ± 0.18 (0.77–1.48)	0.726
Sedentary time (min)	657.23 ± 144.56 (348–1054)	662.73 ± 154.87 (348–1054)	640.73 ± 108.73 (436–846)	0.496
LIPAT (min)	167.32 ± 119.26 (3–520)	174.91 ± 128.93 (3–520)	144.56 ± 81.71 (26–370)	0.254
MIPAT (min)	30.99 ± 51.14 (0–270)	29.07 ± 52.22 (0–269)	36.78 ± 48.22 (0–205)	0.500
VIPAT (min)	-	-	-	-
Mean bouts number (n°)	11.07 ± 13.73 (0–58)	10.60 ± 13.80 (0–57)	12.49 ± 13.66 (0–58)	0.537
B1 (n°)	5.80 ± 6.49 (0–24)	5.66 ± 6.71 (0–24)	6.24 ± 5.88 (0–24)	0.689
B2 (n°)	1.98 ± 2.68 (0–11)	1.87 ± 2.61 (0–11)	2.29 ± 2.93 (0–11)	0.485
B3 (n°)	1.13 ± 1.57 (0–8)	1.04 ± 1.49 (0–5)	1.38 ± 1.79 (0–3)	0.337
B4 (n°)	0.53 ± 0.88 (0–4)	0.55 ± 0.92 (0–4)	0.47 ± 0.73 (0–3)	0.659
B < 5 (n°)	9.44 ± 11.05 (0–46)	9.13 ± 11.15 (0–40)	10.38 ± 10.91 (0–46)	0.613
B^3^5 (n°)	1.63 ± 3.20 (0–17)	1.47 ± 3.25 (0–17)	2.11 ± 3.05 (0–12)	0.370
Lying down (min)	584.33 ± 116.75 (285–893.25)	573.15 ± 125.82 (285–893.25)	617.87 ± 76.24 (492–759.67)	0.085
Sleeping (min)	476.71 ± 109 (255–723)	472.45 ± 116.79 (255–723)	489.47 ± 81.86 (304–641)	0.485
Sleeping efficacy (%)	81.65 ± 9.26 (49–100)	82.43 ± 8.87 (53–100)	79.32 ± 10.16 (49–93)	0.131
TSH (mU/mL)	1.91 ± 1.48 (0.18–7.98)	1.93 ± 1.47 (0.18–7.98)	1.85 ± 1.53 (0.73–7.96)	0.820
fT3 (pg/mL)	2.79 ± 0.36 (2.05–3.91)	2.76 ± 0.35 (2.05–3.91)	2.88 ± 0.39 (2.06–3.63)	0.152
fT4 (ng/dL)	0.83 ± 0.18 (0.48–1.31)	0.83 ± 0.19 (0.48–1.31)	0.84 ± 0.16 (0.59–1.20)	0.691
C-reactive protein (mg/dL)	0.69 ± 1.04 (0.02–6.31)	0.69 ± 1.06 (0.02–6.31)	0.69 ± 1.02 (0.03–4.92)	0.996
Cardiovascular diseases (n° no/n° yes)	46/62	37/44	9/18	0.368
Cerebrovascular diseases (n° no/n° yes)	75/33	57/24	18/9	0.614
Respiratory diseases (n° no/n° yes)	87/21	63/18	24/3	0.545
Oncological diseases (n° no/n° yes)	43/65	31/50	12/15	0.495
Articular pain (n° no/n° yes)	40/68	28/53	12/15	0.350

Data concerning continuous variables are presented as mean ± SD. Minimum and maximum value of each variable are presented, italicized, in brackets. Data concerning categorical variables are presented as n° no/n° yes. Statistical significances concerning the comparison of continuous variables are referring to the Student’s *t*-test results. Statistical significances concerning the comparison of categorical variables are referring to the chi-square test results. Abbreviations: FMI, Fat mass index; FFMI, Free-fat mass index; MMI, Muscle mass index; METs, metabolic equivalents; LIPAT, low intensity physical activity; MIPAT, moderate intensity physical activity; VIPAT, vigorous intensity physical activity; -, unrecorded data; BX, number of daily bouts of moderate intensity physical activity lasting X consecutive minutes.

**Table 2:** Significant correlations of thyroid hormones with physical activity and sleeping parameters, adjusting for the effects of age, CRP and MMI.

	TSH (mU/mL)	fT3 (pg/mL)	fT4 (ng/dL)	METs	Sedentary Time (min)	LIPAT (min)	MIPAT (min)	Mean Bouts Number (n°)	B1 (n°)	B2 (n°)	B3 (n°)	B4 (n°)	B < 5 (n°)	B ≥ 5 (n°)	Lying Down (min)	Sleeping (min)	Sleeping Efficacy (%)
TSH (mU/mL)	1	-	−0.293 **	0.187 °	-	-	-	-	-	-	-	0.213 *	-	0.179 °	−0.242 *	-	-
fT3 (pg/mL)		1	-	-	-	-	-	-	-	-	-	-	-	-	-	-	-
fT4 (ng/dL)			1	−0.227 *	-	−0.202 *	−0.173 °	-	-	-	-	-	-	−0.177 °	0.233 *	0.188 *	-

CRP, c-reactive protein; MMI, muscle mass index; METs, metabolic equivalents; LIPAT, low intensity physical activity; MIPAT, moderate intensity physical activity; BX, number of daily bouts of moderate intensity physical activity lasting X consecutive minutes; ° 0.05 < *p* < 0.1; * 0.05 ≤ *p* < 0.01; ** *p* ≤ 0.01; -, non-significant.

**Table 3:** Comparisons of sub-samples characteristics, according to TSH tertile memberships, using the one-way analysis of variance and Bonferroni test, as post hoc analysis.

Characteristics	TSH 1st Tertile (TSH < 1.11 mU/mL) (n = 35)	TSH 2nd Tertile (1.11 ≤ TSH ≤ 2.12 mU/mL) (n = 38)	TSH 3rd Tertile (TSH > 2.12 mU/mL) (n = 35)	*p*
Age (years)	92.77 ± 3.14	92.21 ± 3.02	93.60 ± 3.32	0.175
Waist circumference (cm)	95.54 ± 10.66	95.58 ± 10.28	96.49 ± 11.48	0.927
Hip circumference (cm)	101.71 ± 8.81	101.31 ± 11.33	103.34 ± 9.75	0.670
Waist to Hip ratio	0.93 ± 0.06	0.94 ± 0.07	0.93 ± 0.07	0.902
FMI (kg of fat mass/m^2^)	6.67 ± 3.46	6.50 ± 3.86	7.14 ± 3.74	0.747
FFMI (kg of fat-free mass/m^2^)	17.98 ± 2.71	18.84 ± 2.76	18.69 ± 3.08	0.398
MMI (kg of muscle mass/m^2^)	11.20 ± 3.04	11.91 ± 2.45	11.82 ± 2.93	0.504
METs (METs)	0.95 ± 0.14 ^	1.03 ± 0.20	1.06 ± 0.25 ^	0.066
Sedentary time (min)	668.97 ± 131.09	648.29 ± 156.53	655.20 ± 147.25	0.828
LIPAT (min)	137.37 ± 98.26	183.21 ± 139.03	180.03 ± 112.75	0.195
MIPAT (min)	15.96 ± 20.93 ^	29.58 ± 32.37	47.57 ± 78.33 ^	0.033
VIPAT (min)	-	-	-	-
Mean bouts number (n°)	7.37 ± 8.67	12.27 ± 11.27	13.47 ± 18.92	0.142
B1 (n°)	4.22 ± 4.79	6.94 ± 6.33	6.16 ± 7.88	0.190
B2 (n°)	1.45 ± 2.25	2.18 ± 2.52	2.29 ± 3.20	0.372
B3 (n°)	0.77 ± 1.10	1.25 ± 1.23	1.36 ± 2.16	0.239
B4 (n°)	0.33 ± 0.55	0.47 ± 0.62	0.80 ± 1.26	0.071
B<5 (n°)	6.77 ± 7.99	10.83 ± 9.87	10.61 ± 14.27	0.222
B^3^5 (n°)	0.59 ± 1.04 ^	1.44 ± 2.09	2.86 ± 4.87 ^	0.010
Lying down (min)	617.66 ± 127.89	578.86 ± 116.15	556.95 ± 99.54	0.085
Sleeping (min)	501.68 ± 108.39	470.27 ± 118.97	458.72 ± 96.02	0.234
Sleeping efficacy (%)	81.46 ± 8.30	81.16 ± 11.14	82.39 ± 8.07	0.843
TSH (mU/mL)	0.7 ± 0.26 ^	1.58 ± 0.34 ^	3.49 ± 1.57 ^	<0.001
fT3 (pg/mL)	2.89 ± 0.35	2.77 ± 0.30	2.72 ± 0.42	0.139
fT4 (ng/dL)	0.88 ± 0.18 ^	0.86 ± 0.17 ^^	0.75 ± 0.17 ^^,^ ^^	0.005
C-reactive protein (mg/dL)	0.86 ± 1.32	0.48 ± 0.55	0.76 ± 1.13	0.279

Data are presented as mean ± SD. ^ values significantly different according to post hoc analysis, *p* < 0.025 ^^ values significantly different according to post hoc analysis, *p* < 0.025. Abbreviations: FMI, Fat mass index; FFMI, Free-fat mass index; MMI, Muscle mass index; METs, metabolic equivalents; LIPAT, low intensity physical activity; MIPAT, moderate intensity physical activity; VIPAT vigorous intensity physical activity; BX, number of daily bouts of moderate intensity physical activity lasting X consecutive minutes.

**Table 4:** Significant correlations of thyroid hormones with physical activity and sleeping parameters, adjusting for the effects of age, CRP and MMI in female sub-sample.

	TSH (mU/mL)	fT3 (pg/mL)	fT4 (ng/dL)	METs	Sedentary Time (min)	LIPAT (min)	MIPAT (min)	Mean Bouts Number (n°)	B1 (n°)	B2 (n°)	B3 (n°)	B4 (n°)	B < 5 (n°)	B ≥ 5 (n°)	Lying Down (min)	Sleeping (min)	Sleeping Efficacy (%)
TSH (mU/mL)	1	-	−0.255 *	0.226 *	-	-	0.226 *	0.204 °	-	-	0.194 °	0.245 *	-	0.242 *	-0.202°	-	-
fT3 (pg/mL)		1	-	0.229 *	-	-	-	-	-	-	-	-	-	-	-	-	-
fT4 (ng/dL)			1	−0.296 *	-	−0.231 *	−0.244 *	-	-	-	-	-	-	−0.271 *	0.259 *	0.207 °	-

CRP, c-reactive protein; MMI, muscle mass index; METs, metabolic equivalents; LIPAT, low intensity physical activity; MIPAT, moderate intensity physical activity; BX, number of daily bouts of moderate intensity physical activity lasting X consecutive minutes; ° 0.05 < *p* < 0.1; * 0.05 ≤ *p* < 0.01; -, non-significant.

**Table 5:** Comparisons of sub-samples characteristics, according to TSH tertile memberships, using the one-way analysis of variance and Bonferroni test as post hoc analysis, in the female sub-sample.

Characteristics	TSH 1st Tertile (TSH < 1.11 mU/mL) (n = 27)	TSH 2nd Tertile (1.11 ≤ TSH ≤ 2.12 mU/mL) (n = 26)	TSH 3rd Tertile (TSH > 2.12 mU/mL) (n = 28)	*p*
Age (years)	93.19 ± 3.37	92.54 ± 3.31	93.46 ± 3.28	0.582
Waist circumference (cm)	95.33 ± 11.29	92.58 ± 13.03	93.43 ± 9.64	0.671
Hip circumference (cm)	101.33 ± 9.27	99.92 ± 12.94	101.57 ± 9.61	0.837
Waist to Hip ratio	0.94 ± 0.06	0.92 ± 0.07	0.92 ± 0.07	0.652
FMI (kg of fat mass/m^2^)	7.36 ± 3.45	7.58 ± 4	7.36 ± 4	0.970
FFMI (kg of fat-free mass/m^2^)	17.44 ± 2.31	18.12 ± 2.69	17.82 ± 2.55	0.621
MMI (kg of muscle mass//m^2^)	10.56 ± 2.59	11.57 ± 2.41	11.02 ± 2.17	0.318
METs (METs)	0.92 ± 0.11 ^	1.03 ± 0.24	1.10 ± 0.24 ^	0.010
Sedentary time (min)	685.37 ± 129.87	667.68 ± 182.82	636.30 ± 150.38	0.498
LIPAT (min)	120.20 ± 86.53 ^^,^ ^^	205.37 ± 161.30 ^^,^ ^^	199.38 ± 115.93 ^	0.024
MIPAT (min)	12.32 ± 16.44 ^	22.64 ± 25.91	51.19 ± 79.82 ^	0.015
VIPAT (min)	-	-	-	-
Mean bouts number (n°)	6.39 ± 8.04	11.08 ± 11.43	14.20 ± 18.71	0.108
B1 (n°)	3.67 ± 4.51	6.90 ± 7.08	6.42 ± 7.84	0.163
B2 (n°)	1.35 ± 2.32	1.85 ± 2.31	2.40 ± 3.08	0.339
B3 (n°)	0.71 ± 1.08	1.03 ± 1.18	1.38 ± 1.98	0.253
B4 (n°)	0.32 ± 0.57 ^	0.41 ± 0.58	0.90 ± 1.31 ^	0.042
B < 5 (n°)	6.06 ± 7.69	10.20 ± 10.46	11.10 ± 13.98	0.207
B^3^5 (n°)	0.34 ± 0.55 ^	0.89 ± 1.40	3.09 ± 4.98 ^	0.003
Lying down (min)	622.08 ± 140.28 ^	544.24 ± 116.57	552.83 ± 108.45 ^	0.043
Sleeping (min)	500.98 ± 122.67	456.14 ± 126.09	460.09 ± 99.88	0.300
Sleeping efficacy (%)	80.64 ± 8.86	83.34 ± 10.49	83.32 ± 7.14	0.442
TSH (mU/mL)	0.64 ± 0.27 ^	1.69 ± 0.34 ^	3.39 ± 1.52 ^	<0.001
fT3 (pg/mL)	2.85 ± 0.36	2.75 ± 0.30	2.68 ± 0.38	0.204
fT4 (ng/dL)	0.89 ± 0.21 ^	0.84 ± 0.17	0.75 ± 0.18 ^	0.034
C-reactive protein (mg/dL)	0.99 ± 1.48	0.44 ± 0.48	0.64 ± 0.92	0.163

Data are presented as mean ± SD. ^ values significantly different according to post hoc analysis, *p* < 0.025, ^^ values significantly different according to post hoc analysis, *p* < 0.025 Abbreviations: FMI, Fat mass index; FFMI, Free-fat mass index; MMI, Muscle mass index; METs, metabolic equivalents; LIPAT, low intensity physical activity; MIPAT, moderate intensity physical activity; VIPAT vigorous intensity physical activity; BX, number of daily bouts of moderate intensity physical activity lasting X consecutive minutes.

## Data Availability

The data are available under reasonable request.

## References

[1] Bowers J., Terrien J., Clerget-Froidevaux M., Gothié J.D., Rozing M.P., Westendorp R.G.J., Van Heemst D., Demeneix B.A. (2013). Thyroid Hormone Signaling and Homeostasis During Aging. Endocr. Rev..

[2] Franceschi C., Ostan R., Mariotti S., Monti D., Vitale G. (2019). The Aging Thyroid: A Reappraisal Within the Geroscience Integrated Perspective. Endocr. Rev..

[3] Hoogendoorn E.H., Hermus A.R., de Vegt F., Ross H.A., Verbeek A.L., Kiemeney L.A., Swinkels D.W., Sweep F.C., Heijer M.D. (2006). Thyroid Function and Prevalence of Anti-Thyroperoxidase Antibodies in a Population with Borderline Sufficient Iodine Intake: Influences of Age and Sex. Clin. Chem..

[4] Moon J.H., Park Y.J., Kim T.H., Han J.W., Choi S.H., Lim S., Park D.J., Kim K.W., Jang H.C. (2014). Lower-But-Normal Serum TSH level Is Associated with the Development or Progression of Cognitive Impairment in Elderly: Korean Longitudinal Study on Health and Aging (KLoSHA). J. Clin. Endocrinol. Metab..

[5] Guan H., Shan Z., Teng X., Li Y., Teng D., Jin Y., Yu X., Fan C., Chong W., Yang F. (2008). Influence of iodine on the reference interval of TSH and the optimal interval of TSH: Results of a follow-up study in areas with different iodine intakes. Clin. Endocrinol..

[6] Chaker L., Korevaar T.I., Medici M., Uitterlinden A.G., Hofman A., Dehghan A., Franco O.H., Peeters R.P. (2016). Thyroid Function Characteristics and Determinants: The Rotterdam Study. Thyroid.

[7] Boucai L., Surks M.I. (2009). Reference limits of serum TSH and free T4 are significantly influenced by race and age in an urban outpatient medical practice. Clin. Endocrinol..

[8] Lago-Sampedro A.M., Gutiérrez-Repiso C., Valdés S., Maldonado C., Colomo N., Almaraz M.C., Rubio-Martín E., Morcillo S., Esteva I., de Adana M.S.R. (2015). Changes in thyroid function with age: Results from the Pizarra population-based longitudinal study. Int. J. Clin. Pract..

[9] Bremner A.P., Feddema P., Leedman P.J., Brown S.J., Beilby J.P., Lim E.M., Wilson S.G., O’Leary P.C., Walsh J.P. (2012). Age-related changes in thyroid function: A longitudinal study of a community-based cohort. J. Clin. Endocrinol. Metab..

[10] Strich D., Karavani G., Edri S., Chay C., Gillis D. (2017). Ft3 is Higher in Males than in Females and Decreases Over the Lifespan. Endocr. Pract..

[11] Tabatabaie V., Surks M.I. (2013). The aging thyroid. Curr. Opin. Endocrinol. Diabetes Obes..

[12] Sell M.A., Schott M., Tharandt L., Cissewski K., Scherbaum W.A., Willenberg H.S. (2008). Functional central hypothyroidism in the elderly. Aging Clin. Exp. Res..

[13] Waring A.C., Arnold A.M., Newman A.B., Bùzková P., Hirsch C., Cappola A.R. (2012). Longitudinal changes in thyroid function in the oldest old and survival: The cardiovascular health study all-stars study. J. Clin. Endocrinol. Metab..

[14] Mariotti S., Franceschi C., Cossarizza A., Pinchera A. (1995). The aging thyroid. Endocr. Rev..

[15] Strich D., Karavani G., Edri S., Gillis D. (2016). TSH enhancement of FT4 to FT3 conversion is age dependent. Eur. J. Endocrinol..

[16] Cappola A.R., Arnold A.M., Wulczyn K., Carlson M., Robbins J., Psaty B.M. (2015). Thyroid Function in the Euthyroid Range and Adverse Outcomes in Older Adults. J. Clin. Endocrinol. Metab..

[17] Ceresini G., Marina M., Lauretani F., Maggio M., Bandinelli S., Ceda G.P., Ferrucci L. (2016). Relationship Between Circulating Thyroid-Stimulating Hormone, Free Thyroxine, and Free Triiodothyronine Concentrations and 9-Year Mortality in Euthyroid Elderly Adults. J. Am. Geriatr. Soc..

[18] van de Ven A.C., Netea-Maier R.T., de Vegt F., Ross H.A., Sweep F.C., Kiemeney L.A., Smit J.W., Hermus A.R., den Heijer M. (2014). Associations between thyroid function and mortality: The influence of age. Eur. J. Endocrinol..

[19] Altay S., Onat A., Can G., Tusun E., Şimşek B., Kaya A. (2018). High-normal thyroid-stimulating hormone in euthyroid subjects is associated with risk of mortality and composite disease endpoint only in women. Arch. Med. Sci..

[20] Pereg D., Tirosh A., Elis A., Neuman Y., Mosseri M., Segev D., Lishner M., Hermoni D. (2012). Mortality and Coronary Heart Disease in Euthyroid Patients. Am. J. Med..

[21] Ogliari G., Smit R.A.J., van der Spoel E., Mari D., Torresani E., Felicetta I., Lucchi T.A., Rossi P.D., van Heemst D., de Craen A.J.M. (2016). Thyroid Status and Mortality Risk in Older Adults with Normal Thyrotropin: Sex Differences in the Milan Geriatrics 75+ Cohort Study. J. Gerontol. Ser. A.

[22] Boucai L., Hollowell J.G., Surks M.I. (2011). An Approach for Development of Age-, Gender-, and Ethnicity-Specific Thyrotropin Reference Limits. Thyroid.

[23] Yeap B.B., Alfonso H., Hankey G.J., Flicker L., Golledge J., Norman P.E., Chubb S.A.P. (2013). Higher free thyroxine levels are associated with all-cause mortality in euthyroid older men: The Health in Men Study. Eur. J. Endocrinol..

[24] van Vliet N.A., van der Spoel E., Beekman M., Slagboom P.E., Blauw G.J., Gussekloo J., Westendorp R.G.J., van Heemst D. (2017). Thyroid status and mortality in nonagenarians from long-lived families and the general population. Aging.

[25] Zhang Y., Chang Y., Ryu S., Cho J., Lee W.-Y., Rhee E.-J., Kwon M.-J., Pastor-Barriuso R., Rampal S., Han W.K. (2014). Thyroid Hormones and Mortality Risk in Euthyroid Individuals: The Kangbuk Samsung Health Study. J. Clin. Endocrinol. Metab..

[26] Veronese N., Fernando-Watutantrige S., Maggi S., Noale M., Stubbs B., Incalzi R.A., Zambon S., Corti M.C., Perissinotto E., Crepaldi G. (2017). Serum Thyroid-Stimulating Hormone Levels and Frailty in the Elderly: The Progetto Veneto Anziani Study. Rejuvenation Res..

[27] Leader A., Ayzenfeld R.H., Lishner M., Cohen E., Segev D., Hermoni D. (2014). Thyrotropin Levels within the Lower Normal Range Are Associated with an Increased Risk of Hip Fractures in Euthyroid Women, but Not Men, over the Age of 65 Years. J. Clin. Endocrinol. Metab..

[28] Ostan R., Monti D., Mari D., Arosio B., Gentilini D., Ferri E., Passarino G., De Rango F., D’Aquila P., Mariotti S. (2018). Heterogeneity of Thyroid Function and Impact of Peripheral Thyroxine Deiodination in Centenarians and Semi-Supercentenarians: Association with Functional Status and Mortality. J. Gerontol. Ser. A.

[29] van den Beld A.W., Visser T.J., Feelders R.A., Grobbee D.E., Lamberts S.W. (2005). Thyroid hormone concentrations, disease, physical function, and mortality in elderly men. J. Clin. Endocrinol. Metab..

[30] Di Iorio A., Paganelli R., Abate M., Barassi G., Ireland A., Macchi C., Molino-Lova R., Cecchi F. (2021). Thyroid hormone signaling is associated with physical performance, muscle mass, and strength in a cohort of oldest-old: Results from the Mugello study. Geroscience.

[31] Ceresini G., Marina M., Lauretani F., Maggio M., Serra M.F., Meschi T., Bandinelli S., Ceda G.P., Ferrucci L. (2018). Physical performance across the thyroid function values within the normal range in adult and older persons. Aging.

[32] Landi F., Abbatecola A.M., Provinciali M., Corsonello A., Bustacchini S., Manigrasso L., Cherubini A., Bernabei R., Lattanzio F. (2010). Moving against frailty: Does physical activity matter?. Biogerontology.

[33] Atzmon G., Barzilai N., Hollowell J.G., Surks M.I., Gabriely I. (2009). Extreme Longevity Is Associated with Increased Serum Thyrotropin. J. Clin. Endocrinol. Metab..

[34] Molino-Lova R., Sofi F., Pasquini G., Gori A., Vannetti F., Abbate R., Gensini G.F., Macchi C. (2013). The Mugello Study, a survey of nonagenarians living in Tuscany: Design, methods and participants’ general characteristics. Eur. J. Intern. Med..

[35] Newman A.B., Siscovick D.S., Manolio T.A., Polak J., Fried L.P., Borhani N.O., Wolfson S.K. (1993). Ankle-arm index as a marker of atherosclerosis in the Cardiovascular Health Study. Cardiovascular Heart Study (CHS) Collaborative Research Group. Circulation.

[36] Calabró M.A., Welk G.J., Eisenmann J.C. (2009). Validation of the SenseWear Pro Armband Algorithms in Children. Med. Sci. Sports Exerc..

[37] Ridley K., Ainsworth B.E., Olds T.S. (2008). Development of a Compendium of Energy Expenditures for Youth. Int. J. Behav. Nutr. Phys. Act..

[38] Rich C., Geraci M., Griffiths L., Sera F., Dezateux C., Cortina-Borja M. (2013). Quality Control Methods in Accelerometer Data Processing: Defining Minimum Wear Time. PLoS ONE.

[39] Schutz Y., Kyle U., Pichard C. (2002). Fat-free mass index and fat mass index percentiles in Caucasians aged 18–98 y. Int. J. Obes..

[40] Bull F.C., Al-Ansari S.S., Biddle S., Borodulin K., Buman M.P., Cardon G., Carty C., Chaput J.-P., Chastin S., Chou R. (2020). World Health Organization 2020 guidelines on physical activity and sedentary behaviour. Br. J. Sports Med..

[41] Wennberg P., Boraxbekk C.-J., Wheeler M., Howard B., Dempsey P., Lambert G., Eikelis N., Larsen R., Sethi P., Occleston J. (2016). Acute effects of breaking up prolonged sitting on fatigue and cognition: A pilot study. BMJ Open.

[42] Sardinha L.B., Ekelund U., dos Santos L., Cyrino E., Silva A.M., Santos D.A. (2015). Breaking-up sedentary time is associated with impairment in activities of daily living. Exp. Gerontol..

[43] Hackney A.C., Saeidi A. (2019). The thyroid axis, prolactin, and exercise in humans. Curr. Opin. Endocr. Metab. Res..

[44] Roa Dueñas O.H., Koolhaas C., Voortman T., Franco O.H., Ikram M.A., Peeters R.P., Chaker L. (2021). Thyroid Function and Physical Activity: A Population-Based Cohort Study. Thyroid.

[45] Stel V.S., Smit J.H., Pluijm S., Visser M., Deeg D.J., Lips P. (2004). Comparison of the LASA Physical Activity Questionnaire with a 7-day diary and pedometer. J. Clin. Epidemiol..

[46] Ravaglia G., Forti P., Maioli F., Pratelli L., Vettori C., Bastagli L., Mariani E., Facchini A., Cucinotta D. (2001). Regular moderate intensity physical activity and blood concentrations of endogenous anabolic hormones and thyroid hormones in aging men. Mech. Ageing Dev..

[47] Olivieri A., Andò S., Bagnasco M., Meringolo D., Mian C., Moleti M., Puxeddu E., Regalbuto C., Taccaliti A., Tanda M.L. (2019). The iodine nutritional status in the Italian population: Data from the Italian National Observatory for Monitoring Iodine Prophylaxis (OSNAMI) (period 2015–2019). Am. J. Clin. Nutr..

[48] Leko M.B., Gunjača I., Pleić N., Zemunik T. (2021). Environmental Factors Affecting Thyroid-Stimulating Hormone and Thyroid Hormone Levels. Int. J. Mol. Sci..

